# Path to net zero is critical to climate outcome

**DOI:** 10.1038/s41598-021-01639-y

**Published:** 2021-11-12

**Authors:** Tianyi Sun, Ilissa B. Ocko, Elizabeth Sturcken, Steven P. Hamburg

**Affiliations:** Environmental Defense Fund, New York City, NY 10010 USA

**Keywords:** Climate-change mitigation, Projection and prediction

## Abstract

Net zero greenhouse gas targets have become a central element for climate action. However, most company and government pledges focus on the year that net zero is reached, with limited awareness of how critical the emissions pathway is in determining the climate outcome in both the near- and long-term. Here we show that different pathways of carbon dioxide and methane—the most prominent long-lived and short-lived greenhouse gases, respectively—can lead to nearly 0.4 °C of warming difference in midcentury and potential overshoot of the 2 °C target, even if they technically reach global net zero greenhouse gas emissions in 2050. While all paths achieve the Paris Agreement temperature goals in the long-term, there is still a 0.2 °C difference by end-of-century. We find that early action to reduce both emissions of carbon dioxide and methane simultaneously leads to the best climate outcomes over all timescales. We therefore recommend that companies and countries supplement net zero targets with a two-basket set of interim milestones to ensure that early action is taken for both carbon dioxide and methane. A one-basket approach, such as the standard format for Nationally Determined Contributions, is not sufficient because it can lead to a delay in methane mitigation.

## Introduction

The concept of net zero greenhouse gas (GHG) emissions is now a central element of government and business commitments to address climate change, with more net zero policies and pledges being rolled out on an almost daily basis. However, discussions continue to focus on the year in which net zero is achieved as the determinant of a successful climate outcome. Rather, the path to net zero is equally as important as when it is achieved, because different paths yield different climate outcomes especially in the near-term.

**Figure 1:**
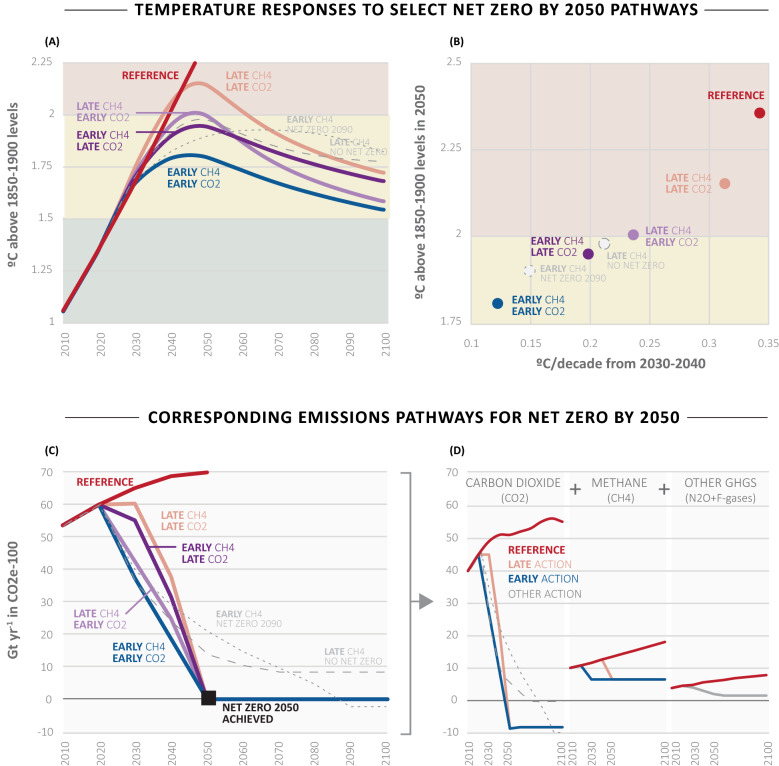
Global emissions pathways and corresponding temperature outcomes discussed in this study. (**A**) Global mean temperature increases relative to the pre-industrial (1850–1900) period resulting from various global emissions pathways shown in (**C**,**D**). (**B**) The near-term (2030–2040) rate of warming and mid-term (2050) temperature increase corresponding to (**A**). (**C**) Global GHG emissions pathways (Gt CO_2_e-100; calculated using Global Warming Potentials with a 100-year time horizon^1^) used in this study. (**D**) The breakdown of carbon dioxide, methane, and other GHG emissions (nitrous oxide and fluorinated gases) in (**C**). Colored solid lines and markers indicate pathways that meet the net zero GHG emissions by 2050 target. Gray dashed lines and markers indicate pathways that do not meet the net zero GHG emissions by 2050 target.

Here we show that pathways that include early action to reduce emissions of both carbon dioxide and methane (Fig. 1, panel A)—the two most prominent long- and short-lived GHGs, respectively^1,2^—yields the lowest temperature outcomes over all timescales. We therefore recommend that countries and companies adopt supplementary interim milestones in addition to net zero targets that encourage early emissions reductions of both of these gases. This can be pursued by following a two-basket approach that identifies a specific near-term milestone for each gas.

Beyond the increased likelihood of achieving long-term temperature targets, three potential advantages of this supplemental two-basket milestone strategy include: (i) slowing down the rate of warming in the near-term, (ii) lowering the mid-century peak warming, and (iii) reducing dependence on negative emissions technologies in the medium to long-term. The success of the two-basket milestone strategy ultimately depends on countries and companies committing to and achieving ambitious targets in both baskets. However, this strategy is particularly beneficial relative to a one-basket because it encourages separate treatment of long- and short-lived GHGs, and thereby circumvents the many challenges of using a metric to compare GHGs with vastly different lifetimes^3^. For example, Global Warming Potential with a 100-year time horizon (GWP100)—the most commonly employed metric to aggregate emissions in a one-basket approach—downplays the importance of short-lived GHG mitigation to avoided warming in the following few decades, which can therefore lead to missed opportunities to maximize climate benefits before midcentury^3^.

Awareness of the importance of the path and action to address this issue are critical given that the number of net zero pledges has doubled in less than a year. This includes more than 1,500 companies with a combined revenue of more than $11.4 trillion^4^ and more than 20 national governments^5^ currently accounting for at least one third of global GHG emissions^6^. Several more governments are currently considering active legislation to adopt targets^7^, and investors are urging companies to adopt net zero goals and prepare for a zero-emissions economy^8^.

Net zero targets, in particular net zero carbon dioxide emissions, is a critical element for achieving the Paris Agreement temperature goals^9–11^. Previous studies have discussed many aspects of net zero with regards to its application in policy, including the definitions of relevant terminology, timeline difference between net zero carbon and net zero GHG emissions, near-term emissions reductions and mitigation investments, and impact of climate metrics on the net zero timeline^12–16^. Studies have also identified multiple shortcomings of current net zero targets, and called for strategies for improvement. Some of those issues are a need for transparency relating to types of GHGs covered and metrics used; consistency in the accounting method for land-use emissions; clarity in definitions and terminology; distinction among carbon dioxide removals, reductions and avoidance; consideration of fairness regarding different timelines for achieving net zero among countries with a diversity of economic conditions; and concern over companies/countries using the long timeline of net zero targets to delay decarbonization^13,17–21^. Proposals to improve net zero targets include but are not limited to transparency in the scope of emissions regarding gas species, sources, and metrics; disclosure of contributions from emissions reductions, removals, and offsets; disclosure of fairness and adequacy of target timeline as required in the Paris Agreement Nationally Determined Contributions (NDCs); long-term roadmap of maintaining net zero or net negative emissions; and plans to monitor and manage carbon storage.

Further, current net zero targets do not inherently call for early action on short-lived GHGs, which a growing body of research shows is a key strategy to slow down warming in the near-term^22–26^. Emissions of short-lived GHGs account for nearly a third of today’s gross warming^27^, and given that they don’t last long in the atmosphere, emissions reductions in these GHGs can quickly lead to slowing down the global-mean rate of warming. Short-lived GHGs’ role in net zero commitments can be undervalued and misunderstood, due in part to the metrics issue described above, which can lead to missed opportunities to lower damages in the near- (2021–2040) to mid-term (2041–2060).

Our recommended approach of supplementing net zero commitments with separate interim milestones for methane and carbon dioxide emissions builds on previous recommendations discussed above^13,17–21^ and the two-basket approach to GHG mitigation that has been proposed by earlier studies [e.g.^28,29^] but not widely adopted. This strategy can help constrain the emissions pathway and encourage early action for both short- and long-lived GHGs, thereby strengthening the probability of meeting globally agreed upon temperature goals while reducing the damages suffered in the interim.

## Common misconceptions of net zero targets

Net zero GHG targets are considered the primary strategy to achieve long-term temperature goals. The current net zero framework rose to prominence as a result of analyses reported by the Intergovernmental Panel on Climate Change (IPCC)^9^ as well as language in the Paris Agreement^11^ that suggested the net zero concept and suitable timelines consistent with global temperature targets. While variations in the definition exist^14,15,30^, current net zero pledges most often use the definition adopted by the United Nations Framework Convention on Climate Change (UNFCCC): a point in time (typically around 2050) when no further GHGs are being added to the atmosphere through human activities beyond what can be removed by human interventions^11^. However, as previous reports and studies have noted, the net zero concept is far more complex than widely perceived and many aspects of net zero and the Paris Agreement are open for interpretation^4,15,31,32^. As a result, several misconceptions have evolved that in some cases can threaten the effectiveness of targets.

First, there have been misinterpretations about the ideal timeline; the timeline identified by the IPCC for achieving net zero *carbon dioxide* emissions consistent with temperature targets has been largely interpreted as the timeline for achieving net zero *greenhouse gas* emissions, but the time period for the latter is decades later^9^. Net zero by 2050 is also the strictest timeline within the flexibility given by Article 4 of the Paris Agreement that outlines net zero GHGs to be achieved “in the second half of this century”^11^. However, it is widely perceived by the general public and environmental organizations that achieving net zero greenhouse gas emissions by 2050 is a requirement by the Paris Agreement^33–36^. While achieving net zero GHG emissions as early as possible is generally better for the climate, it can potentially deter commitments if companies and countries become overwhelmed with the aggressive timeline. Therefore, it is important that decision makers are aware that 2050 is not a “required” timeline for net zero GHG emissions, rather that net zero would likely occur before 2100 to achieve temperature goals^15^.

Second, there has been confusion over the role of short-lived GHGs in net zero targets^4,37^; combining all GHG emissions into one target obfuscates the different actions needed for short- versus long-lived GHGs (most prominently methane and carbon dioxide, respectively). For example, while we need to prevent the build-up of long-lived GHGs in the atmosphere via net zero emissions (and thus adhere to a budget)^38^, short-lived GHG emissions don’t need to reach zero, only have their rates reduced to not contribute to additional warming^9,39^. The emphasis on a combined net zero target can therefore lead to a lack of attention to cumulative emissions for long-lived GHGs, and a misguided perception that short-lived GHGs need to reach zero, or more commonly, that net negative carbon dioxide emissions must compensate for residual short-lived GHG emissions. Rather, we can still achieve climate stabilization with residual non-zero emissions of short-lived GHGs that are not compensated for by negative carbon dioxide emissions as long as their emission rate is gradually declining over time, because these pollutants do not build up in the atmosphere over long time periods^9,39^.

And third, many do not realize that net zero emissions goals do not address the climate crisis over all timescales, only long-term warming and climate stabilization. This is consistent with the temperature goals of the Paris Agreement, which is to stay below certain levels of warming in the long-term, but also means that net zero targets in isolation are not designed to slow down the rate of warming in the next few decades which would reduce additional damages from increases in temperature. Further, the aggregation of GHG emissions, using GWP100 as required by the UNFCCC, makes it difficult to unambiguously determine the climate impact of the Paris Agreement emission goal of “peaking of GHG emissions as soon as possible” and undertaking “rapid reductions thereafter”^11^, because the breakdown between short- and long-lived GHG emissions is unknown; for example one could increase short-lived GHG emissions but decrease long-lived GHG emissions, with a net decrease that suggests a peak and decline, but more warming in the following few decades. Given that effective strategies exist to reduce near-term climate damages^25,26^, it is important that climate policies pursue these actions as well as those focused on stabilizing the climate. Together, cumulative damages can be reduced relative to those incurred from focusing on only one of these objectives.

In addition to these impactful misunderstandings^4, 33–37^, the critical role of the emissions pathway in determining the climate outcome is not widely understood—threatening anticipated benefits of climate action as well as potentially missing opportunities to further reduce near-term damages. There is a perception that the date net zero is achieved is the sole indicator of success^32,36^, yet different paths yield different outcomes and thus damages. Therefore, the net zero framing can unintentionally yield a false sense of what is needed to stay below agreed upon temperature goals because some paths can overshoot temperature goals. Further, there is a false sense of rigid requirements^33–37^, because it is also possible to achieve temperature goals even if humanity does not succeed in meeting the net zero goals set out (Fig. 1; gray lines). And finally, the role of early action in limiting damage in the near-term is largely hidden in the net zero construct.

To clarify the importance of the path to minimizing climate damages, we examined a range of GHG emissions pathways, all of which achieve net zero by 2050 and yet result in a range of temperature outcomes (Fig. 1; colored lines and markers). Some are far better than others at lowering the rate of warming over the next few decades, some experience peak warming temperatures well below and even above 2 °C, and there are different end-of-century warming levels.

## The role of the net zero path in determining climate outcomes

We assess four illustrative mitigation pathways that encompass a range of possibilities for achieving global net zero GHG emissions by 2050 (the most ambitious timeline consistent with the Paris Agreement). Each path is considered feasible given existing technologies and/or consistent with policy discussions. For example, abatement potentials for methane, nitrous oxide, and fluorinated (F)-gases are constrained by technological feasibility and availability of effective mitigation strategies, and for carbon dioxide are constrained by the ultimate goal of achieving net zero by 2050 (the average timeline for emissions pathways consistent with 1.5 °C according to IPCC^9^). Emissions of aerosols and reactive gases are assumed to follow the average of Shared Socio-economic Pathways that reach similar radiative forcing levels as the Representative Concentration Pathway 2.6 (Supplementary Fig. 1)^40,41^. The mitigation pathways of these pollutants are influenced by decarbonization and air quality policy that is not discussed in this study, but the sensitivity of our results to three different levels of mitigation is tested (Supplementary Fig. 2). Overall, the reduction of aerosol and reactive gas emissions tends to increase warming rate in the immediate term before the benefit of reducing GHGs starts to dominate, and increase peak warming in all scenarios. Nonetheless, the benefit of early action on major GHGs is consistent across different levels of aerosol and reactive gas emissions. See “Methods” section for more details.

We consider two mitigation timelines for the two most prominent GHGs, carbon dioxide and methane: ‘early’ and ‘late’ action. We focus on these two gases because they account for over 70% of today’s positive radiative forcing from GHG emissions to date, and they also represent the dominant long-lived and short-lived GHGs, respectively, for both current and future warming in the absence of climate action^42^. While the paths we consider are generally illustrative, they are within the range of emissions pathways projected by integrated assessment models^9^ or considered technically achievable^25,26,46,47^. However, actual carbon dioxide and methane reductions over time can take many forms, and socio-economic constraints and large-scale deployment of various technologies, such as negative emissions technologies, will play a major role in determining exact paths. For the purpose of this analysis, we do not vary nor explore these variables further.

For carbon dioxide, we are constrained by paths that achieve net zero emissions in 2050. Therefore, in this study, carbon dioxide emissions are reduced following a linear path and only reach the amount of net negative emissions needed to compensate for residual non-carbon dioxide emissions mathematically. Early action for carbon dioxide includes immediately reducing emissions, at a rate of 18 gigatonnes per year (Gt/yr) emissions every decade from 2020 (45Gt/yr) to 2050 (-8Gt/yr), and late action includes keeping the 2020 emissions level (45Gt/yr) through 2030 then quickly reducing to net negative emissions in 2050. Achieving -8Gt/yr of net carbon dioxide emissions in 2050 is within the range of scenarios estimated by the integrated assessment models used in the IPCC 2018 report^9^. While net negative carbon dioxide emissions generally increase in magnitude toward 2100 in these scenarios, we keep it constant at -8Gt/yr from 2050 to 2100 for simplicity.

For methane, recent studies have shown that 45–55% of methane emissions reductions by 2030 can be considered achievable with existing technologies and strategies^25,26^. Therefore in this analysis, early action for methane includes roughly halving methane emissions by 2030 (relative to the reference scenario of a projected 383 million metric tonnes per year (MMt/yr) based on Ocko et al.^25^) then keeping a constant emission rate at 200 MMt/yr throughout the century. Late action includes taking no action until 2040 and then reducing to 200 MMt/yr by 2050.

Combinations of these early and late mitigation timelines for carbon dioxide and methane, along with mitigation of nitrous oxide and F-gases, make up the four pathways: (1) early action for both carbon dioxide and methane; (2) early action for carbon dioxide and late action for methane; (3) late action for carbon dioxide and early action for methane; and (4) late action for both carbon dioxide and methane (see Methods for more details on the model and pathways). Each pathway achieves net zero GHG emissions in 2050 using the standard metric for accounting under the UNFCCC: Carbon Dioxide Equivalence (CO_2_e) using GWP100 for non-CO_2_ gases.

### Impact of path on key climate indicators

We use the reduced-complexity climate model MAGICC^43^ to assess global mean temperature responses to each pathway, and analyze key climate change indicators: rate of warming, which is associated with the pace of damages and the ability for society and ecosystems to adapt to changes; peak warming, which is associated with loss of some ecosystems and tipping point thresholds; and long-term warming, which is associated with shifts in biomes and sea level rise^9^. Results are shown in Fig. 1 and described below:

- **Rate of warming**—Early methane action has the largest impact on slowing the rate of warming over the next few decades. When combined with early carbon dioxide mitigation, the slowdown is maximized. If early action only applies to carbon dioxide emissions, there is an appreciable slowdown in the rate of warming, but not nearly as much as with methane or methane alone (Fig. 1; note that we restrict the magnitude of early carbon dioxide reductions to a reasonably realistic path, which constrains the degree to which the rate of warming can slow down). If we delay reductions in emissions of both GHGs relative to early reductions in both, the rate of warming is close to the reference emissions pathway for at least a couple decades, meaning we miss a powerful opportunity to limit social and environmental damages in the near-term.
- **Peak warming**—Peak warming is lowest for the case with early action on both GHGs, and can be considered “well below 2 °C.” The early methane action and late carbon dioxide action scenario has a lower peak warming than early carbon dioxide action and late methane action; this is because peak warming occurs by midcentury, and methane plays an outsized role in near-term warming due to its potent yet short-lived characteristics. Therefore, delaying methane action leads to a larger peak warming around midcentury. Further, early action for both compared to late action for both can shave 0.4 °C off the peak warming, and late action for both can even temporarily breach the 2 °C temperature target despite meeting the net zero by 2050 target (Fig. 1, panel B).
- **Long-term warming**—Despite emissions pathways being nearly identical post-2050, end-of-century warming varies considerably for the four emissions scenarios (Fig. 1, panels A and C). This is mostly due to the amount of carbon dioxide emitted before 2050. Given that carbon dioxide is a long-lived GHG, the more carbon dioxide we emit before the net zero target is achieved, the more carbon dioxide there is in the atmosphere for centuries to come—committing the planet to warming for generations. Therefore, early action prevents a considerable amount of carbon dioxide from ever entering the atmosphere (i.e. a smaller carbon budget), that would otherwise have to be removed at a later date to achieve similar long-term temperature outcomes. Further, although the early methane action and late carbon dioxide action scenario shows a lower peak warming than vice versa, it has a higher long-term warming level, because more carbon dioxide has built up in the atmosphere throughout this path. However, it is important to note that in all four cases, the level of warming at end-of-century can be considered “well below 2 °C.” Further, for early action for both gases, the temperature drops close to 1.5 °C. While none of the illustrative pathways in our analysis reach temperature below 1.5 °C by 2100, it does not represent the full range of climate futures. For example, if net negative carbon dioxide emissions were to grow in magnitude toward 2100 as carbon capture technologies scale up, it is possible to reach temperature below 1.5 °C by 2100^9^.

### The importance of early action

Early action to mitigate both carbon dioxide and methane is clearly beneficial for reducing climate damages over all timescales relative to other mitigation timelines. It yields the lowest rate of warming, peak warming, and long-term warming. It also helps to avoid temporarily overshooting the 2 °C temperature goal of which the consequences are not well understood^9^. An additional benefit of early carbon dioxide and methane action (which includes actions to protect carbon stocks such as stopping tropical deforestation) is that we are less dependent on nascent, currently expensive, or as-of-yet unavailable carbon dioxide removal (CDR) technologies, such as large-scale direct air capture of carbon dioxide. While the investment in advancing CDR technologies is important, a greater focus on mitigating short-lived GHGs along with decarbonization by companies and countries could reduce dependence on CDR to achieve climate goals.

### The outsized role of methane in the near-term

Standard climate metrics (i.e. CO_2_e-100 and GWP100) downplay the impact of short-lived GHGs—such as methane—on warming in the next few decades. For example, the methane reductions (Fig. 1D) appear modest relative to the carbon dioxide reductions, but they have a substantial impact on the near-term warming rate. The reason the reductions appear modest is because methane emissions are being valued based on their cumulative radiative impacts over 100 years, which undervalues methane’s true radiative impact relative to carbon dioxide over shorter time horizons (< 30 years). This highlights a key analytical issue with net zero commitments, and why researchers have called for metrics that better convey climate impacts of short-lived GHGs over all timescales^3,39,44^. In fact, the 10 MMt CO_2_e/yr of methane emitted in 2010 had a similar impact on warming over the following 10 years compared to the 40 MMt CO_2_/yr in 2010 based on the latest understanding of methane’s direct and indirect radiative effects^2^. This is partly why cutting methane emissions can drive down the rate of warming more quickly than cutting carbon dioxide emissions.

### Failing to achieve net zero by 2050

It is important to note that while midcentury targets are useful for getting companies and governments to commit to ambitious action, net zero GHG emissions by 2050 is neither a required timeline for achieving temperature targets^45^ nor a cliff foretelling failure^16^. We could potentially miss net zero by 2050 targets and still succeed at staying below temperature goals, if we act on methane and never exceed the maximum carbon budget allowed to stay below 2 °C (Fig. 1; gray lines and markers). For example, emissions pathways that achieve net zero GHG emissions in 2090 (dotted gray line) or do not achieve net zero GHG emissions at all (dash gray line) can be consistent with staying below temperature targets throughout this century. Note that both pathways do achieve net zero carbon dioxide emissions well before 2100, and the one with early methane reduction (dotted gray line) has a larger maximum carbon budget for similar end-of-century temperature outcomes. This is not to suggest we abandon net zero by 2050 targets, but rather to highlight the advantages of accelerating progress by exploiting components currently receiving much less attention and investment, including rapid reductions in methane emissions and preventing tropical deforestation, and thus greatly increase the probability of achieving humanity’s collective temperature goals. These opportunities largely arise from the ability to aggressively address methane emissions as a separate goal^46,47^ while continuing a sharp focus on carbon dioxide emissions reductions.

## Two-basket interim milestones for net zero targets

While the simplicity of the 2050 net zero goal has led to successfully mobilizing companies and governments to commit to ambitious climate actions on all GHG emissions, it has also de-emphasized the nuanced role of the pathway relative to the conceptually easy point-in-time target. Given that early action leads to significantly more climate benefits over all timescales than pathways with later action, we recommend that companies and countries supplement the original point-in-time net zero targets with a two-basket set of interim milestones to ensure early action, for both short- and long-lived GHGs separately (most notably methane and carbon dioxide, respectively). The adoption of these supplementary interim milestones would retain the simplicity and familiarity of the net zero concept while bringing critical details to the forefront that are key to achieving commonly held climate goals.

The interim milestones would include two near-term targets between now and the net zero year, one for short-lived (e.g. methane) and one for long-lived (e.g. carbon dioxide) GHGs (i.e. a two-basket approach) in addition to the multi-GHG ‘net zero by a specific year’ target. At the country level, this approach is distinct from the standard Paris Agreement NDCs—which can be considered interim milestones for longer-term net zero goals—because those are often one-basket, in that there is just one target that generally includes all GHGs. However, a country can submit these two-basket milestones as part of their NDCs.

A two-basket approach for interim milestones—as opposed to a one-basket interim milestone—is critical because reducing short- and long-lived GHGs benefit the climate over different timescales, and we could miss out on better outcomes in both the near- and long-term if the gases are combined into one target^28,29^. The standard metric for combining GHGs (CO_2_e-100) also misrepresents the climate impacts of early action for short-lived GHGs, which further threatens realizing significant climate benefits. While the role of methane and short-lived GHGs mitigation has been investigated by many studies^22,25,48–53^, it has been debated whether the additional benefit of a separate mitigation policy is significant^23,45,54–56^. Some previous studies suggested that early actions to mitigate GHG emissions should focus on CO_2_, if there is competition between CO_2_ and methane abatement measures [e.g.^57–60^]. However, there is growing evidence of and attention to the benefits of early methane action^25,26,61^, and given the many affordable measures available for distinct emissions sources^25^, there is strong support to act early for both CO_2_ and methane. Our analysis shows a clear benefit of early and rapid versus late methane mitigation in near-term rate of warming, which would likely not occur without an explicit methane policy^47,62^. Further, a two-basket approach to emissions accounting can also increase the effectiveness of international emission offset agreements by avoiding additional climate damages from emission offsets with imperfect climate metrics^63^ and reduce the uncertainties in climate impacts with emissions trading^64^.

Overall, without two-basket interim milestones, the emissions pathway to net zero can take various forms^16^. Depending on different combinations of the amount, type, and timing of GHGs emitted before net zero, it is both possible to remain well below or overshoot temperature goals even if the global community reaches net zero by 2050 (Fig. 1; colored lines). These insights are largely hidden to most companies and policymakers, and a two-basket interim milestone approach would bring them to the forefront and increase our chance of a better climate outcome over all timescales.

We need to exploit our growing understanding of the options we have in addressing climate change to ensure an effective, equitable, and rapid outcome. Acting early on methane and carbon dioxide would limit warming over all timescales, maximize reductions in the rate of warming, and make achieving our goals more likely by making the path forward more affordable and less dependent on technology not yet available at scale.

## Methods

The global mean temperature change is simulated by the freely available reduced complexity climate model, Model for the Assessment of Greenhouse-gas Induced Climate Change (MAGICC) version 6^43^. MAGICC6 consists of an upwelling-diffusion ocean coupled to a four-box atmosphere and a globally averaged carbon cycle model. The model parameters are calibrated against the more sophisticated Coupled Model Intercomparison Project CMIP3 atmosphere–ocean and C4MIP carbon cycle models. It can reliably simulate the impact of GHG emissions on climate without relying on much computational resource. In this analysis, the model is run using mostly the default properties and medium parameters including 3 °C climate sensitivity. The methane-related properties are updated based on the latest research, including radiative efficiency^65^, atmospheric chemical lifetime^1^, and tropospheric ozone radiative efficiency^66^ (see Data S1).

For all the experiments, the model is run from 1765 to 2100 with historical GHG concentrations and prescribed aerosol forcing before 2005 and emissions of gases and aerosols from 2005 and onward. The global mean temperature increase simulated by MAGICC6 in 2010–2019 is 1.19 °C relative to the 1850–1900 level, within the *likely* range of 0.8–1.3 °C assessed by the latest IPCC report and higher than the best estimate of 1.07 °C^27^. It is important to note that a single model projection with one set of parameters cannot represent the full range of possible future temperatures. However, this paper focuses on the differences between emission pathways rather than the exact outcome of a particular pathway.

Two groups of experiments were conducted: The first group contains four emission pathways that reach the target of net zero GHG emissions in 2050; the second group contains one emission pathway that reaches net zero GHG emissions in 2090 and one pathway that does not reach net zero GHG emissions at all. All emission pathways reach net zero CO_2_ emissions well before 2100. Only CO_2_ and methane emissions pathways are different among these experiments. The CO_2_ mitigation pathways are constrained by the need to achieve net zero around mid-century and previous estimates^14,45,67^. Two illustrative pathways of CO_2_ emissions are used for the experiments where the net zero GHG emissions by 2050 target is achieved—early action and late action. Early action is represented by reducing enough CO_2_ emissions this decade to be on track to achieve the negative emissions (-8Gt/yr) needed in 2050. Late action is represented by keeping the same emissions rate till 2030 and taking drastic measures to achieve negative emissions in 2050. In the experiments where global emissions do not reach net zero by 2050, CO_2_ emissions are constrained by the corresponding methane emission pathway and the goal of limiting temperature below 2 °C. The methane mitigation pathways are constrained by existing technologies^25,46,47^ and also characterized by early and late action. Early action is represented by halving methane emissions by 2030 relative to the reference scenario (200 MMt/yr) then held constant thru 2100. Late action is represented by following the reference scenario emissions until 2040 then reducing to 200 MMt/yr by 2050.

Nitrous oxide (N_2_O) and Fluorinated gases emissions follow a scenario consistent with limiting warming below 2 °C^9,68^. N_2_O is slowly reduced to ~ 70% of the 2010 level by mid-century and held constant thru 2100. This mitigation pathway is consistent with the limited and uncertain mitigation potential for N_2_O^9^ and can be considered as early action since emissions start to reduce immediately. All species of fluorinated (F-)gases are slowly phased out by 2060 with emission levels reduced to 94%, 63%, and 25% relative to 2020 level by 2030, 2040, and 2050 respectively, consistent with the Kigali Amendment to the Montreal Protocol^68^. We do not vary the emissions pathways of N_2_O and F-gases in this analysis, because their potential mitigation pathways play a relatively minor role in determining future level of warming compared to carbon dioxide and methane.

Emissions of aerosols and reactive gases are assumed to follow a selected group of combined Shared Socio-economic Pathways and Representative Concentration Pathways (SSP-RCPs). These combined SSP-RCPs are used in the latest Coupled Model Intercomparison Project phase 6 (CMIP6) to take into account both end-of-century radiative forcing levels and socio-economic factors such as population, governance, technology advancements, education, and gross domestic product (GDP)^69^. Because the emissions pathways of aerosols and reactive gases are influenced by both decarbonization and air quality policy, we consider three mitigation levels to show the sensitivity of our results to these emissions (Supplementary Figs. 1 and 2). For the no/low mitigation level, we use the RCP8.5^40^ as in the reference scenario where aerosols and reactive gases are slightly reduced from 2020 to 2100 except for NH_3_ (Supplementary Fig. 1; red lines). For the strong mitigation level, we use the SSP1-1.9 scenario^41^ where stringent air quality policy is applied and emissions are the lowest (orange lines). For the intermediate mitigation level, we use the average of SSPx-2.6 scenarios (x includes SSP1, 2, 4, and 5)^41^ where the radiative forcing level is consistent with the 2 °C temperature goal, which is appropriate for net zero pathways, while assuming no specific air quality policy (yellow lines). The resulting temperature outcomes are shown in Supplementary Fig. 2 and the intermediate mitigation level outcome is shown in Fig. 1. Overall, the reduction of aerosols and reactive gases can increase peak and end-of-century warming by up to 0.2 °C, and increase the rate of warming in the immediate term (Supplementary Fig. 2).

The outcomes of global mean temperature increase from all emission pathways are compared to a reference scenario that corresponds to a world where no additional policies are implemented after what was legislated by the end of 2017^70^.

## Supplementary Information

Supplementary Information 1.

Supplementary Information 2.

## Data Availability

All data are available in the supplementary materials.
