# Author Correction: Path to net zero is critical to climate outcome

**DOI:** 10.1038/s41598-021-02588-2

**Published:** 2021-11-24

**Authors:** Tianyi Sun, Ilissa B. Ocko, Elizabeth Sturcken, Steven P. Hamburg

**Affiliations:** Environmental Defense Fund, New York City, NY 10010 USA

Correction to: *Scientific Reports* 10.1038/s41598-021-01639-y, published online 12 November 2021

The original version of this Article contained an error in the Acknowledgments section.

“We thank Michael Oppenheimer, Suzi Kerr, Kelley Kizzier, Sam Kotis, Jon Coifman, Anne Marie Borrego, Jordan Faires, and Christina Baute for thoughtful feedback and discussions. Two anonymous reviewers provided constructive suggestions that have considerably strengthened the manuscript. This work is supported by the Robertson Foundation, Heising-Simons Foundation, and Klarman Family Foundation.”

now reads:

“We thank Michael Oppenheimer, Suzi Kerr, Kelley Kizzier, Sam Kotis, Jon Coifman, Anne Marie Borrego, Jordan Faires, and Christina Baute for thoughtful feedback and discussions. Two anonymous reviewers provided constructive suggestions that have considerably strengthened the manuscript. This work is supported by the Robertson Foundation and Heising-Simons Foundation.”

The original Article has been corrected.

