# Supplementary material for: Path to net zero is critical to climate outcome
Source: Sci Rep. 2021 Nov 12;11:22173. doi: 10.1038/s41598-021-01639-y (PMC8589977; doi:10.1038/s41598-021-01639-y)
Supplement: Supplementary file 2 — Supplementary Information 2. [file 41598_2021_1639_MOESM2_ESM.pdf]

Supplementary Materials for
Path to net zero is critical to climate outcome

Authors: Tianyi Sun[1], Ilissa B. Ocko[1*], Elizabeth Sturcken[1], Steven P. Hamburg[1]

Affiliations:

[1]Environmental Defense Fund; New York City, New York, 10010, USA.

*Corresponding author. Email: iocko@edf.org

Content:

Supplementary Figures 1-2

Data S1

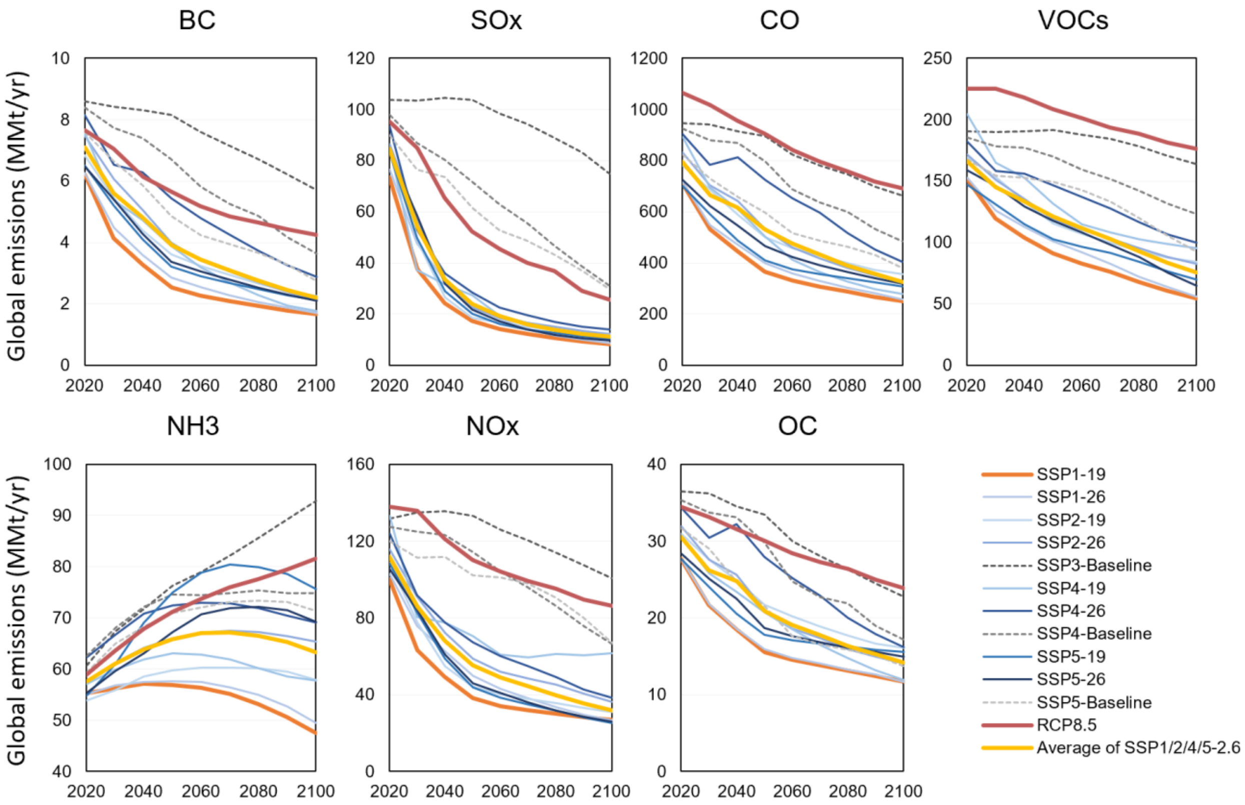

Supplementary Figure 1. Aerosols and reactive gases emissions used in this study compared to SSP-RCPs scenarios. Emissions of black carbon (BC), sulfate aerosols (mainly SO2), carbon monoxide (CO), non-methane volatile organic compounds (VOCs), ammonia (NH3), nitrogen oxides (mainly NO2), and organic carbon (OC) through 2100 in the (red) RCP8.5 scenario, (gray dash lines) SSP3/4/5 baseline scenarios, (blue) SSP1/2/4/5-1.9/2.6 scenarios, (orange) SSP1-1.9 scenario, and (yellow) average of SSP1/2/4/5/-2.6 scenarios. Red, orange, and yellow denote the three levels of mitigation used in the sensitivity test in Supplementary Figure 2. Units are MMt/yr for all emissions.

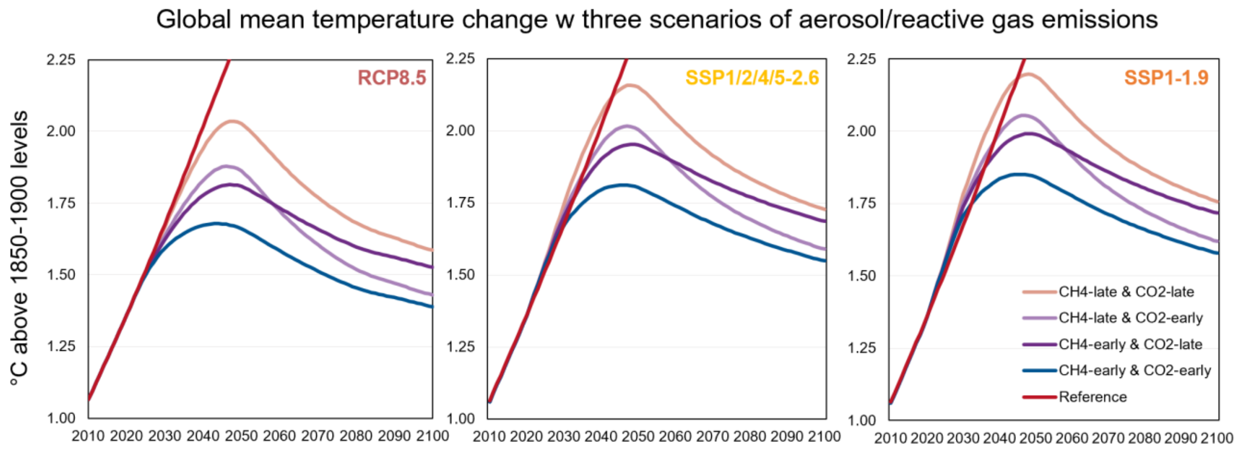

Supplementary Figure 2. Temperature outcomes of net zero pathways under different levels of aerosols and reactive gases mitigation. Global mean temperature outcomes (°C) of four net zero pathways as in Figure 1a but with (left) no/low aerosol and reactive gas mitigation corresponding to red lines in Supplementary Figure 1, (middle) intermediate aerosol and reactive gas mitigation corresponding to yellow lines in Supplementary Figure 1, and (right) strong aerosol and reactive gas mitigation corresponding to orange lines in Supplementary Figure 1.
